# Identifying and Mitigating Adverse X‑ray Induced Effects in Operando Spectroscopic Studies of Copper Exchanged Zeolites

**DOI:** 10.1021/acs.jpcc.5c06740

**Published:** 2025-11-26

**Authors:** Johannes Wieser, Mark A. Newton, Przemyslaw Rzepka, Paul M. Leidinger, Mahesh Ramakrishnan, Justus Just, Jeroen A. van Bokhoven

**Affiliations:** † Department of Chemistry and Applied Biosciences, Institute for Chemical and Bioengineering, 27219 ETH Zurich, 8093 Zürich, Switzerland; ‡ J. Heyrovsky Institute of Physical Chemistry, Czech Academy of Sciences, 182 23 Prague 8, Czech Republic; § Interdisciplinary Nanoscience Center, 1006 Aarhus University, Gustav Wieds Vej 14, 8000 Aarhus C, Denmark; ∥ 226073 MAX IV Laboratory, Fotongatan 2, 224 84 Lund, Sweden; ⊥ Center for Energy and Environmental Science, Paul Scherrer Institute (PSI), 5232 Villigen, Switzerland

## Abstract

The increase in brilliance and global proliferation of synchrotron facilities has established synchrotron radiation as an indispensable tool in modern science. However, as synchrotron sources become more powerful, the risk of X-ray induced alterations becomes increasingly pronounced. These beam-induced effects will compromise results, leading to unusable data at best and incorrect conclusions at worst. Techniques that guarantee that the means of observation do not cause the observed phenomena are therefore an absolute necessity. This work presents a methodology to mitigate beam-induced effects, consisting of performing a mesh scan — in which the beam is rastered across the sample bed — to avoid local overexposure. The validity of this approach is demonstrated using copper-exchanged zeolites — materials frequently investigated at synchrotron facilities — by monitoring the change in oxidation state of copper during exposure to oxidizing, reducing and inert atmospheres via time-resolved X-ray absorption spectroscopy (XAS) as a function of X-ray dose. This method reveals that beam-induced effects can manifest at significantly lower X-ray doses than previously reported for this class of material. More importantly, it provides a blueprint for conducting operando measurements that ensure uncompromised results as well as the full exploitation of the capabilities offered by state-of-the-art fourth generation synchrotron facilities.

## Graphical abstract

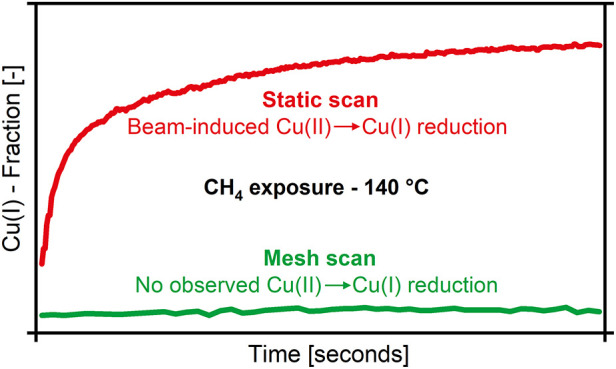

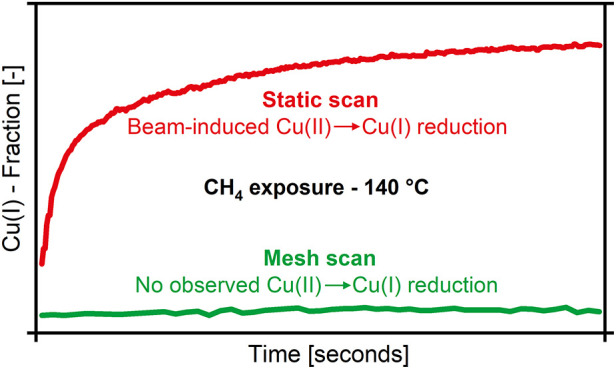

## 1 Introduction

Synchrotron radiation is an essential tool across a diverse array of disciplines, with the potential applications using its radiation constantly evolving and expanding.^1^ Numerous X-ray based techniques, such as X-ray diffraction (XRD), X-ray microscopy and X-ray absorption and emission spectroscopy (XAS and XES) are all commonly used techniques at synchrotron facilities.^2,3^ This broad scope of techniques finds application across a wide spectrum of research disciplines, including material science and catalysis,^2,4^ battery^5^ and fuel cell^6^ research, biochemistry,^7^ environmental science,^8^ but also in areas as diverse as archeology and cultural heritage in general,^9–14^ among many other fields. This expansion of application and opportunities is, in part, due to the global proliferation of synchrotron facilities.^15^ Beyond their increase in number, synchrotrons have continually evolved since their inception, with the fourth generation of synchrotrons having entered operation over the past decade. This evolution has led to an exponential increase in the synchrotron source brightness,^16^ and has enabled a significant increase in the achievable coherence and flux density of the beam, among many other advancements. This in turn has allowed for quicker scan times, smaller beams, and lower limits of detection when compared to previous generation synchrotron facilities.^17,18^ In addition, advances in end-station technology, such as more advanced monochromators, focusing optics, and improvements to detector efficiency, allow for the conducting of more complex experiments. These factors, combined with suitable sample environments and sample presentation, have resulted in real-time in situ and operando investigations becoming routine experimental procedures at synchrotron facilities.^4,19,20^ Especially in fields such as materials chemistry, as well as thermo-, electro- and photocatalysis, in situ and operando synchrotron measurements have rapidly become indispensable tools, enabling the elucidation of structure–performance relationships under relevant reaction conditions in real-time.^21,22^ Changes often occur on short time scales, and monitoring them has become feasible at synchrotrons, with a temporal resolution to the level of picoseconds being achievable.^23^

These significant advancements in both synchrotron sources and end-stations however often come with caveats. The steep increase in achievable flux density, while beneficial for certain applications, has proven detrimental for others. For biological^13,18,24^ and organic materials^13,25,26^ in particular—but certainly not limited to them—beam-induced effects may already arise at significantly less invasive conditions than achievable at state-of-the-art synchrotrons. Consequently, the undesired and damaging effects of prolonged radiation exposure to biological and organic materials in particular have been recognized and understood for decades;^27–29^ photoelectrons may be taken up by the macrostructure,^11,13,29^ solvated electrons and radicals caused by the radiolysis of H_2_O resulting in bond breakage,^12,13,29–33^ or a reduction of metal cations^28,29^ are all distinct possibilities. Such processes can often be slowed down by performing measurements at cryogenic temperatures.^31,34^ Cryogenic cooling may however not be able to entirely avoid beam-induced effects, instead merely slowing them down.^13^ If data integrity is the primary motivation for avoiding any beam-induced effects, cryogenic cooling may be a feasible protection strategy. If the main aim instead is to protect the sample from irreversible beam damage—as will be the case with, for example, cultural artifacts—extreme protective measures as cryogenic cooling may simply be an additional source of damage.^13^ Beyond the potential damaging effects of cryogenic cooling to the sample itself, such an extreme measure for avoiding any beam-induced effects becomes entirely unfeasible for any in situ and operando investigations. Clearly, not every protection strategy will be suitable for every sample and application.

The complexity of the experiment itself will dictate what mitigation strategies can or need to be applied to avoid any beam-induced effects. When therefore exposing materials to—in addition to high degrees of radiation—potentially reactive operating conditions, the situation becomes significantly more complex. When exposing ex situ samples to radiation, the source of any change observed in the data or sample must be caused by the interaction of the beam with the sample. For in situ or operando investigations, the source of change could be, for example, the reactive atmosphere, the beam, the temperature applied, or a combination of these parameters. Not only may these undesired beam-induced effects be exacerbated under such conditions, but entirely new effects may be triggered as well. In situ and operando investigations are therefore prime candidates for the emergence of undesired beam-induced effects. As in biological and organic materials, the potential knock-on effects a given sample may experience by combining a beam of high flux density with reactive atmospheres are diverse; a reduction of metal cations,^25,35–37^ often in tandem with an agglomeration into particles,^38,39^ phase transitions,^18,40,41^ crystal nucleation^42,43^ or the quenching of a reaction^44^ have all been observed. As such, the results obtained when applying powerful X-ray beams may be fundamentally compromised along with any conclusions derived from them. Experiments that require a temporal resolution are especially liable to such effects due to the need to constantly monitor the material via the beam. A typical example of this is an erroneous derivation of the kinetics of a redox reaction due to the X-ray beam causing a metal center to reduce more quickly.^35–37,40,45^ This could at least partially be attributed to a localized heating of the sample due to the beam, meaning that the observed kinetics may stem from a higher local temperature than applied.^45^ If the effect of X-ray illumination would indeed solely be of a thermal nature, the actual chemistry occurring in the system could be assumed to be unaltered. This however will not be the case for all environments. For instance, in a catalytic reaction where H_2_O is either a reactant or a product, species produced by the radiolysis of H_2_O can, instead of just altering the kinetics, change the balance between competing reaction routes or introduce entirely new ones.^45–48^ This will result in a complete misjudgment of the chemistry inherent to the system under study. Standardized techniques to initially detect and consequently avoid beam-induced contributions are therefore a necessity for the exploitation of synchrotron radiation to remain a powerful and versatile tool over such a broad range of applications and user communities.

Copper (Cu) is an element that has been shown to readily reduce when exposed to X-rays.^28,36,45,46,49^ In aqueous solution for example, Cu­(II) reduces to Cu­(I) when exposed to X-ray radiation. In addition, the nature of the Cu-salt employed has shown to influence the degree of Cu reduction as well, with this phenomenon becoming more pronounced in the case that electron-scavenging species were absent.^45,46^ This highlights that the surrounding environment that Cu­(II) finds itself in will significantly affect the ability of beam-induced knock-on effects to result in a reduction to Cu­(I). Beyond aqueous systems, an exposure to a beam of high flux density has also been shown to influence the rate of Cu­(II) reduction to Cu­(I) in Cu-exchanged zeolites (Cu/zeolites) in the presence of CH_4_. Cu/zeolites are studied for both the stepwise^50–52^ and catalytic^53,54^ conversion of CH_4_-to-CH_3_OH (MtM), and are industrially applied in the NH_3_ selective catalytic reduction (NH_3_-SCR) of NO_*x*_ gases.^55,56^ Both the industrial relevance and potential has therefore made Cu/zeolites the subject of extensive research, often using synchrotron radiation. In the case of the stepwise conversion of MtM via Cu/zeolites, the reaction follows a two-electron redox mechanism,^57^ wherein two Cu­(II) cations are reduced to two Cu­(I) cations during CH_4_ exposure.^58^ This results in a bound CH_3_O-group or CH_3_OH, which can be desorbed using H_2_O vapor. H_2_O vapor has additionally been suggested to be able to oxidize Cu­(I) to Cu­(II) in Cu/zeolites,^59^ although this remains a disputed topic.^60^ In any case, Cu­(I) is typically reoxidized to Cu­(II) by exposure to a strong oxidant such as O_2_.^50,57^ A simplified scheme of the reaction progression is depicted in Figure 1a. For a more detailed explanation we refer to our previous work.^50,58,61^

**Figure 1:**
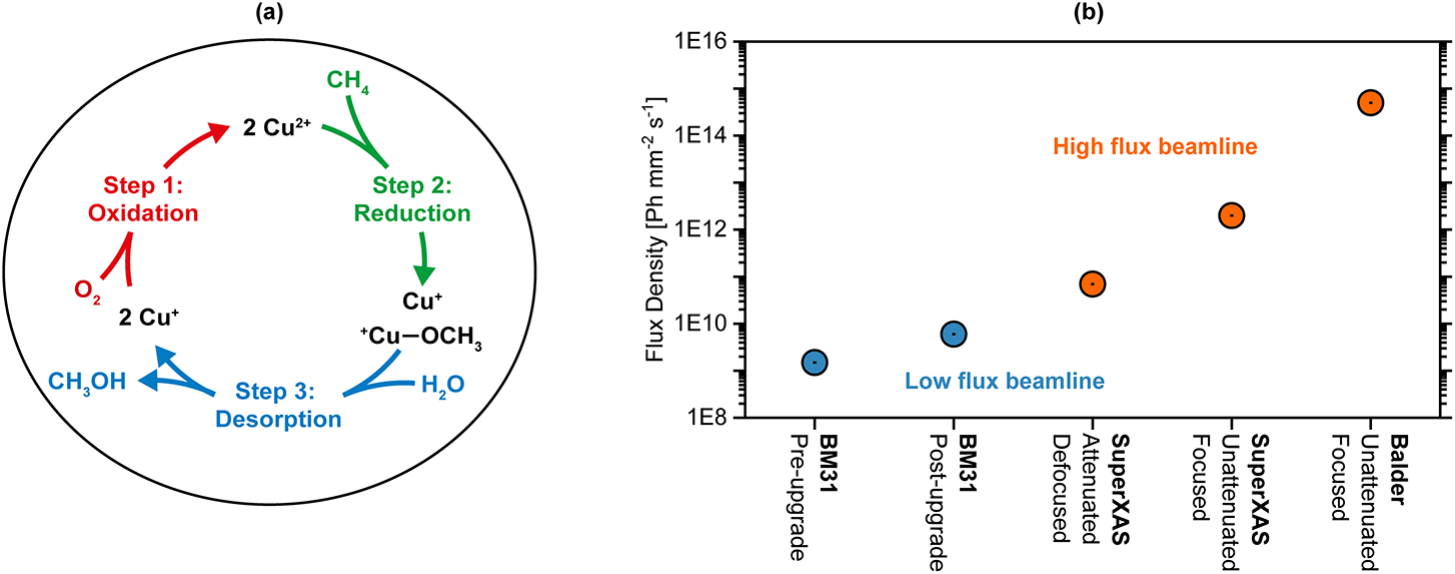
(a) Simplified schematic of the mechanism of the MtM conversion via Cu/zeolites via the oxygen looping approach; (b) comparison of flux densities achievable at ∼9 keV at various beamlines used for investigations into Cu/zeolites for the MtM conversion in past^36^ as well as present studies.

Prior investigations on Cu/zeolites display a clear correlation between increased Cu­(II)-reduction kinetics to Cu­(I) as a function of flux density [Ph mm^–2^ s^–1^].^36^ Therefore, all other factors being equal, the rate of Cu­(II) reduction to Cu­(I) at a high flux spectroscopy beamline (SuperXAS at the Swiss Light Source, Villigen, Switzerland) was shown to be much more rapid than witnessed at a low flux spectroscopy beamline (BM31 of the Swiss-Norwegian Beamlines at the European Synchrotron Radiation Facility, Grenoble, France) for the same Cu/zeolite. However, by defocusing the beam by a factor of 60 and attenuating the beam by 97% at the high flux beamline, similar reaction kinetics were observed. This high degree of attenuation and defocusing at a high flux beamline will however entail a significant loss in data quality when compared to a focused unattenuated beam, leading to, among other drawbacks, a decrease in achievable temporal resolution. These necessary steps to avoid any beam-induced effects therefore negate the potential benefits of using high flux beamlines and state-of-the-art fourth generation synchrotrons. A comparison of the achievable flux densities at both low flux and high flux beamlines at ∼9 keV may be seen in Figure 1b.

Strategies that permit a safe examination of materials while still capitalizing on the advantages offered by high flux densities are essential for ensuring both high data quality and uncompromised results. This work presents a robust strategy that not only minimizes adverse beam-induced effects, but in its execution uncovers previously unknown beam-induced effects. This methodology is exemplified using Cu-exchanged zeolite omega (Cu/omega) examined over the whole range of reactants applied in the scope of the MtM conversion. The extensive knowledge accumulated over decades of research, the broad range of conditions examined, and the separation of the catalytic process into its three distinct stages makes the stepwise MtM conversion via Cu/zeolites an ideal probe reaction for evaluating the effects of elevated flux densities at various conditions.^50,61–64^ The efficacy of the presented strategy is validated by its application at a high flux beamline at the first fourth generation synchrotron (Balder beamline at MAX IV Laboratory, Lund, Sweden). A Comparison with results obtained at a low flux beamline (BM31, ESRF) demonstrates the effectiveness of this method.^36,50^ The technique ensures the collection of scientifically meaningful results while fully leveraging the capabilities of state-of-the-art synchrotron facilities.

## 2 Methods

### 2.1 Material Properties

Zeolite omega was synthesized using an in-house built rotating oven at 110 °C for 20 days.^62,64^ A more detailed description of the synthesis conditions may be found in prior reports.^62–64^ Following the synthesis, the sample was calcined in static air at 550 °C for 8 h (1 K/min ramp rate).^64^ The material was ion-exchanged with 2 M NH_4_NO_3_ solution (50 °C, 24 h), followed by three consecutive ion-exchanges with 0,0025 M Cu­(NO_3_)_2_ solution (50 °C, 24 h). After each exchange the sample was washed with H_2_O and EtOH.

Two batches of Cu/omega were used. The first system exhibits a Si/Al of 4.3 and a Cu loading of 4.4 wt % (Cu/omega-A).^64^ The second system exhibits a Si/Al of 4.3 and 4.7 wt % Cu-loading (Cu/omega-B).^50^ An in-depth examination of both systems has revealed that they behave in a very similar manner under the examined reaction conditions.^50,61^ Cu/omega-B was used for all results depicted in the main text.

### 2.2 Sample Preparation

Prior to examination, both Cu/omega samples were pelletized to 2 tonnes and mortared and sieved to a size of 50–75 μm. Approximately 5–10 mg were loaded into a borosilicate capillary of 1.5 mm diameter. To hold the bed in place, quartz wool was used on either side of the bed.

### 2.3 Experimental Setup

The experiments were carried out at the Balder beamline at the MAX IV Laboratory in Lund, Sweden. A comparison to previous work performed at both BM31 (ESRF) and SuperXAS (SLS) is detailed during this study.^36,50^ Table 1 depicts a summary of the applied conditions in present and prior studies.

**Table 1:** Summary of X-ray Beam Conditions Applied in This Study, as Well as Prior Beam-Damage Studies,^36^ Using the Beam-Induced Reduction of Cu­(II) to Cu­(I) in Cu/Zeolites as a Probe Reaction

abbreviation	beamline/facility	beam size (*H* × *V*)	flux density [Ph mm^–2^ s^–1^]	additional
BM31(1)	BM31/ESRF	4 × 0.5 mm^2^	1.5 × 10^9^	pre EBS upgrade
BM31(2)	BM31/ESRF	4 × 0.5 mm^2^	6.0 × 10^9^	post EBS upgrade
SuperXAS(1)	SuperXAS/SLS	2000 × 150 μm^2^	7.0 × 10^10^	defocused, attenuated
SuperXAS(2)	SuperXAS/SLS	80 × 80 μm^2^	2.0 × 10^12^	focused, attenuated
SuperXAS(3)	SuperXAS/SLS	80 × 80 μm^2^	1.2 × 10^14^	focused, unattenuated
Balder(1)	Balder/MAX IV	75 × 75 μm^2^	6 × 10^11^	attenuated
Balder(2)	Balder/MAX IV	50 × 75 μm^2^	2.8 × 10^13^	unattenuated
Balder(3)	Balder/MAX IV	75 × 75 μm^2^	2.8 × 10^13^	unattenuated

The Balder beamline uses an in-vacuum wiggler, with a maximum flux of about 5 × 10^12^ Ph s^–1^ (monochromatic, with bandwidth of a Si(111) monochromator at 9 keV) being achievable. However, for systems and experiments that do not specifically require such a high flux, it is typically reduced by 1 order of magnitude. At a beam size of typically 75 × 75 μm^2^, this results in a flux density of ∼3 × 10^13^ Ph s^–1^ mm^–2^ at 9 keV. The Balder beamline can perform a mesh grid scan, allowing for multiple points on the sample to be probed in quick succession. A scan was performed between 8785 and 9178.5 eV, alternating the time per scan between one and 10 s.

BM31 is a bending magnet beamline with no focusing optics, causing the beam size to be solely controlled by slits. A flux of ∼3.6 × 10^9^ Ph s^–1^ at 9 keV was achievable pre-upgrade of the ESRF and used for prior beam damage investigations.^36^ The ESRF upgrade^65^ resulted in a 4-fold increase in flux density being achieved at the Cu K-edge, which has been previously applied for monitoring the kinetics of Cu oxidation and reduction in Cu/omega under typical oxygen looping conditions.^50^

The capillary reactor cell is supplied by and available at the Balder beamline and allows for both in situ XAS and XRD data to be collected in quick succession.^66^ The capillary is held in place using PTFE ferrules, with a thermocouple inserted on the outlet side of the capillary. Silicon nitride radiative heaters are used, as well as heat shields with a window large enough to enable collection of XRD data.^66^ A detailed explanation of all other aspects (gas delivery, scrubbing, flow control and switching valves) of the setup, including a graphical representation of the experimental setup, can be found in previous work.^50^ All source gases (He, O_2_, and CH_4_) are fed through moisture traps (Big Universal Trap—RMSH-2—for helium (He) and O_2_, O_2_/Moisture Trap—0T3-2-2S—for CH_4_). Four mass flow controllers are used for four gas flows: O_2_, CH_4_ and two He lines. One He line leads into a home-built (two-stage, variable temperature) bubbler system filled with H_2_O. All lines, connections and the capillary cell inlet and outlets through which H_2_O passes are traced to ∼100 °C using heating lines. Three Valco Vici 4 port switching valves are used to quickly switch between various gas atmospheres. The outlet of the capillary leads to a mass spectrometer for gas composition analysis.

### 2.4 Data Analysis

The XAS data were reduced, in some cases averaged, and normalized using the Prestopronto package.^67^ The linear combination fitting (LCF) GUI of the Prestopronto package was used to examine the copper speciation under reaction conditions. Three internal standards were used; a hydrated Cu­(II) reference, recorded at room temperature, a dehydrated Cu­(II) reference, collected in situ under O_2_ at 450 °C and 1 bar after 30 min of exposure at said temperature, and a Cu­(I) reference collected in situ at 450 °C and 1 bar CH_4_ after 60 min. These three references therefore represent a hydrated and oxidized system (hydrated Cu­(II)), an oxidized and dehydrated system (dehydrated Cu­(II)) and a reduced (Cu­(I)) system. This method has been shown to work well in the case of Cu/omega.^50^ A more detailed explanation of the three internal references used may be found in the Supporting Information.

## 3 Results

### 3.1 Evaluating and Minimizing the Extent of Beam-Induced Reduction Using a Focused Unattenuated Beam

A Cu/omega sample was exposed to the unattenuated and focused beam (Balder(2) & Balder(3)—Table 1) to evaluate the effect of a high flux density on the kinetics of Cu­(II)-reduction to Cu­(I). For context, the flux density where in previous examinations no beam-induced effects were observed is at least 3 orders of magnitude lower (SuperXAS(1) and BM31(1)—Table 1).^36^ Previous examinations focusing on the effect of varying the flux density on the oxidation state of Cu in zeolites applied a temperature of 140 °C under a CH_4_ atmosphere, as the rate of Cu­(II)-reduction to Cu­(I) in Cu/zeolites is negligible under these conditions.^36^ For ease of comparison, the same temperature was chosen. Prior to CH_4_ exposure, all examined materials were exposed to O_2_ at elevated temperatures to sufficiently dehydrate the material, as well as ensure Cu is in an oxidation state of +2.^50^ The results are depicted in Figure 2.

**Figure 2:**
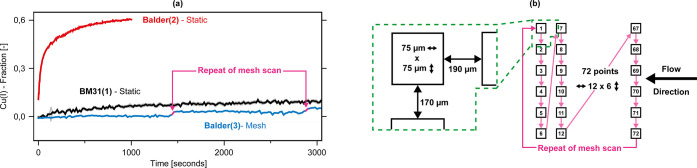
(a) Kinetics of Cu­(I)-formation in Cu/omega with a focused unattenuated beam (Balder(2) & Balder(3)—Table 1) under a CH_4_ atmosphere of 1 bar at 140 °C, using a 2 s scan time. The Cu/omega sample, (Balder(2)—Static, red line) was previously exposed to an O_2_ atmosphere at 300 °C, resulting in primarily dehydrated Cu­(II) being present in the system pre CH_4_ exposure. A second Cu/omega sample (Balder(3)—Mesh, blue line), was exposed to an O_2_ atmosphere at 450 °C before exposing it to CH_4_ at 140 °C. A mesh scan was applied, examining 72 separate spots across the sample bed, as illustrated in (b). The measurement consists of a 2 s scan, followed by a dead-time of 18 s. The pink lines indicate when a mesh scan is repeated, e.g. the movement from the final position, position 72, to the first examined position, position 1, as depicted in (b). As a comparison to conditions where a beam-induced contribution is assumed to be negligible, the change in Cu­(I) fraction of Cu/mordenite at a low flux beamline at 140 °C is depicted as well (BM31(1)—Static, Black line).^36^ The labels correspond to the flux densities applied and listed in Table 1, and if a static scan, e.g. no movement, or a mesh scan, as illustrated in (b), was applied. (b) Schematic representation of the mesh scan applied in the case of Balder(3)—Mesh, depicted in (a). The left portion indicates the beam size, as well as the distance between the examined points. The right side depicts a graphical representation of how the mesh scan is performed. In the case of Balder(3)—Mesh, depicted in (a), 72 separate spots were examined.

Figure 2a depicts the increase in Cu­(I) as a function of time at varying X-ray doses and temperatures under a CH_4_ atmosphere at 1 bar at 140 °C. When applying a focused unattenuated beam (Balder(2)—Table 1), 40% of all Cu in the system has reduced from Cu­(II) to Cu­(I) after ∼300 s (Balder(2)—Static, Figure 2a). This rapid increase in Cu­(I) over such a short time frame stands in contrast to the results obtained at a low flux beamline for Cu/mordenite (BM31(1)—Static, Figure 2a), where the rate of Cu­(II) reduction to Cu­(I) is significantly less pronounced.^36^ The measurement was aborted after ∼1000 s, after ∼60% of the total Cu in Cu/omega was present as Cu­(I). Such a quick initial rate of reduction of Cu­(II) to Cu­(I) in zeolites is typically achieved at temperatures of ∼300 °C, as illustrated in Figure S2a. A movement of 200 μm along the bed after ∼1000 s of CH_4_ exposure to a region previously unexposed to the beam reveals that the Cu­(I) fraction at the unexposed position remains unchanged to values observed prior to CH_4_ exposure (Figure S2b). The first spectrum recorded at the unexposed position after >1000 s under a CH_4_ atmosphere at 140 °C is therefore a validation that under these conditions the reduction of Cu­(II) to Cu­(I) for Cu/omega in actuality progresses very slowly, if at all, when unilluminated. These results clearly show that applying such a high flux density to Cu­(II)/zeolites under a CH_4_ atmosphere causes a significant increase in the rate of reduction of Cu­(II) to Cu­(I), thereby rendering the collected data meaningless for describing the intrinsic behavior of the system under the examined conditions.

A fresh Cu/omega sample was loaded into the reactor system and heated to 450 °C under an O_2_ flow. The higher temperature used in comparison to the previous experiment was chosen to guarantee a complete dehydration of the system.^61^ The temperature was then decreased to 140 °C, and following a He purge, the sample was exposed to CH_4_. As in the case of the previous measurement (Balder(2)—Static, Figure 2a), a scan time of 2 s was chosen. By incorporating a fast-shutter in the measurement sequence, a further 18 s of dead-time for each scan was included to avoid an unnecessary exposure of the sample to the beam. One measurement scan therefore equates to 20 s. The measurements were performed using a mesh scan, thereby examining multiple points across the sample bed in series. Recent literature has suggested that a similar method might be able to mitigate any beam-induced effects on a given sample.^68^ A schematic representation is depicted in Figure 2b. As depicted in Figure 2a, applying such a mesh scan results in the final Cu­(I) fraction not exceeding the level of Cu­(I) observed for Cu/mordenite at a low flux beamline (Balder(3)—Mesh vs BM31(1)—Static, Figure 2a) over the same timeframes.

When the mesh sequence is repeated (movement from pos. 72 to pos. 1, Figure 2b) at ∼1450 s and ∼2900 s, as indicated by the pink lines in Figure 2a, a step-like increase in the Cu­(I) fraction occurs. The regions where the Cu­(I) fraction remains constant, e.g. from zero to 1450 s, correspond to a single mesh sequence of 72 separate points. These findings once again highlight that in absence of the beam, a reduction of Cu­(II) to Cu­(I) in Cu/omega at 140 °C does not seem to occur at all. When, however, the same point is examined repeatedly, the step-like increase is observed. Despite these step-like increases the overall degree of beam-induced reduction remains negligible, as the final values remain below the Cu­(I) fraction obtained at the low flux beamline (BM31(1)—Static, Figure 2a) over the same timeframes. This could lead one to believe that even at the comparatively low flux density applied at the low flux beamline (BM31(1)—Table 1), the employed flux density may already be high enough to cause the observed reduction of Cu­(II) to Cu­(I). When however comparing the derived values for the apparent activation energy (*E*_app_) for Cu/mordenite at a low flux beamline (BM31(1)—Table 1)—and a high flux beamline using a defocused and attenuated beam (SuperXAS(1)—Table 1)^36^—to the *E*_app_ derived for certain Cu active sites from comparably less invasive in situ UV–vis measurements,^51,69^ the values are in good agreement with each other. This would therefore suggest that the measurements conducted with Cu/mordenite at these flux densities (BM31(1) & SuperXAS(1)—Table 1) indeed reflect the actual chemistry occurring in the system. Cu/zeolites are notorious for being able to exhibit a variety of Cu active site configurations, and which active site, or combination of active sites, are present in a given Cu/zeolite is dependent on a multitude of factors, not least zeolite topology and Cu-loading.^58,69,70^ That these factors cause this variation in observed Cu­(II) reducibility in past and present measurements between zeolite mordenite and zeolite omega, as depicted in Figure 3, may therefore be an entirely reasonable explanation as well. Nevertheless, the results indicate that to be certain that any beam-induced contributions are entirely avoided, a given spot should not be examined more than once.

**Figure 3:**
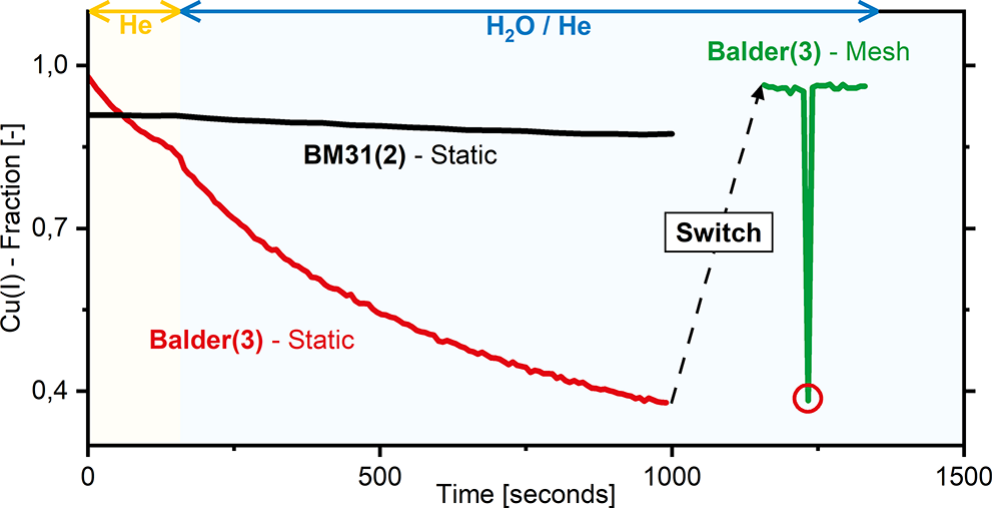
Change in the Cu­(I) fraction as a function of time for Cu/omega. For the first 150 s the samples were exposed to He, followed by the additional introduction of 30 mbar of H_2_O vapor to the feed. A static scan was applied in the case of Balder(3)—Static (red line). Following the termination of the static scan, a mesh scan over the same sample bed was conducted, labeled Balder(3)—Mesh. When applying the mesh scan, the spot previously illuminated during the static scan was also examined, indicated by a red circle. A temperature of 290 °C was chosen. As a comparison to conditions where a beam-induced contribution was assumed to be negligible, the change in the Cu­(I) fraction of Cu/omega at a low flux beamline at 300 °C is depicted as well (BM31(2)—Static, black line). The labels correspond to the flux densities applied and are listed in Table 1.

### 3.2 Evaluating the Extent of Beam Induced Effects under He and H_2_O Vapor Exposure

Within the scope of Cu/zeolites used for the MtM conversion, CH_4_ exposure is followed by H_2_O vapor exposure to desorb the target product CH_3_OH.^58^ The role of H_2_O may however be more nuanced than solely being an extraction medium for CH_3_OH. H_2_O has additionally been suggested to be able to reoxidize certain Cu­(I) configurations to Cu­(II) in Cu/zeolites, albeit not to the same degree as conventional oxidants such as O_2_, as well as with significantly reduced rates.^59^ This claim has however proven to be very contentious, with other works instead observing no anaerobic Cu­(I) to Cu­(II) oxidation for the same zeolite topology at similar conditions.^60^ To investigate if beam exposure might result in a change in the chemistry occurring in the system, we exposed Cu/omega to H_2_O vapor at various flux densities to see if an effect on the rate of Cu­(I) oxidation to Cu­(II) could be observed. The results are depicted in Figure 3.

Figure 3 shows the change in the Cu­(I) fraction as a function of time. All samples were initially exposed to a He atmosphere for 150 s. In the case of the measurements conducted at the low flux beamline (BM31(2), Figure 3), the Cu­(I) fraction remains unchanged. The constant Cu­(I) fraction suggests that the beam is not affecting the chemistry of the system. However, in the case of the static scan applied at a high flux beamline (Balder(3)—Static, Figure 3), the Cu­(I) fraction instead rapidly decreases. These findings highlight that prolonged exposure to a beam of high flux density may even alter the chemistry of the Cu­(I)/zeolite without the addition of reactive gases such as CH_4_, but may already manifest under inert gas exposure.^36^ A potential source could be X-ray irradiation of adsorbed species formed during the Cu/zeolite’s previous reaction with CH_4_ leading to oxidizing species, resulting in an oxidation Cu­(I) to Cu­(II).

He exposure is followed by the additional introduction of H_2_O vapor (30 mbar) to the reactor feed. While under He no change in the Cu­(I) fraction was observed, the Cu­(I) fraction starts decreasing when H_2_O vapor is added to the feed (BM31(2)—Static, Figure 3), albeit at a very low rate. A change in the rate of decrease of the Cu­(I) fraction when switching from pure He to additionally H_2_O vapor when applying a high flux density and a static scan (Balder(3)—Static, Figure 3) suggests that the interaction of the X-rays with H_2_O is at least partially responsible for the observed decrease in Cu­(I) fraction. To further validate that the observed changes are in fact due to the continued illumination of the sample by the beam, a mesh scan (Balder(3)—Mesh) was performed following the static scan (Balder(3)—Static), as depicted in Figure 3. Conducting a mesh scan across the entire sample bed, following the static scan illuminating solely one point, highlights that any significant change in the Cu­(I) fraction is entirely due to beam-induced effects. A red circle indicates when the mesh scan has reached the spot previously illuminated during the static scan, this being the sole spot with such a low Cu­(I) fraction observed during the mesh scan. The lack of any meaningful oxidation of Cu­(I) to Cu­(II) when a mesh scan is applied (Balder(3)—Mesh, Figure 3) suggests that any anaerobic oxidation witnessed at the lower flux density (BM31(2), Figure 3) is beam-induced.

While the observed decrease in the Cu­(I) fraction at a low flux density (BM31(2)—Static, Figure 3), and the absence of a decrease when applying a mesh scan (Balder(3)—Mesh, Figure 3), may be a strong indication for beam induced effects already emerging at lower flux densities during H_2_O vapor exposure, the situation is more complex. When comparing the rate of change of the Cu­(I) fraction of both static scans (BM31(2)—Static and Balder(3)—Static, Figure 3), a difference in the kinetic behavior of the two is observable. The Cu­(I)-fraction at a low flux density (BM31(2)—Static, Figure 3) decreases in a linear manner, while at higher flux densities (Balder(3)—Static, Figure 3), the decrease is nonlinear. This variation may indicate that in both cases different processes are responsible for the observed oxidation of Cu­(I) to Cu­(II). In addition, when examining a spot previously unexposed to the beam following the static scan at high flux densities, a minor decrease in the Cu­(I) fraction may be observed as well, as illustrated in Figure 3. The mesh scan applied following the static scan depicted in Figure 3 reveals that the Cu­(I) fraction across the bed has marginally decreased (by 2%) over ∼800 s of H_2_O vapor exposure. At low flux densities (BM31(2)—Static, Figure 3) the Cu­(I) fraction decreased by 6% over the same timeframes. Definite conclusions about which flux density induces an onset of significant beam-induced contributions over the examined timeframes to the oxidation of a Cu­(I)/zeolite via H_2_O vapor therefore call for further investigations. However, the enormous decrease of the Cu­(I) fraction by 61% witnessed when applying the static scan at high flux densities (Balder(3)—Static, Figure 3) is ample evidence that such beam-induced effects will arise over a prolonged period of X-ray illumination.

## 4 Discussion

### 4.1 The Influence of the Applied Flux Density on the Nature and Magnitude of Beam-Induced Effects

The results of past^36^ and present studies highlight that not only the applied flux density governs whether beam-induced effects will be observed for a given sample, but that the conditions the sample is exposed to will play a deciding role as well. Figure 4 summarizes if beam-induced effects have been observed for past^36^ and present studies, for both reactions examined—the reduction of Cu­(II) to Cu­(I) under a CH_4_ atmosphere, as well as the oxidation of Cu­(I) to Cu­(II) during H_2_O vapor exposure.

**Figure 4:**
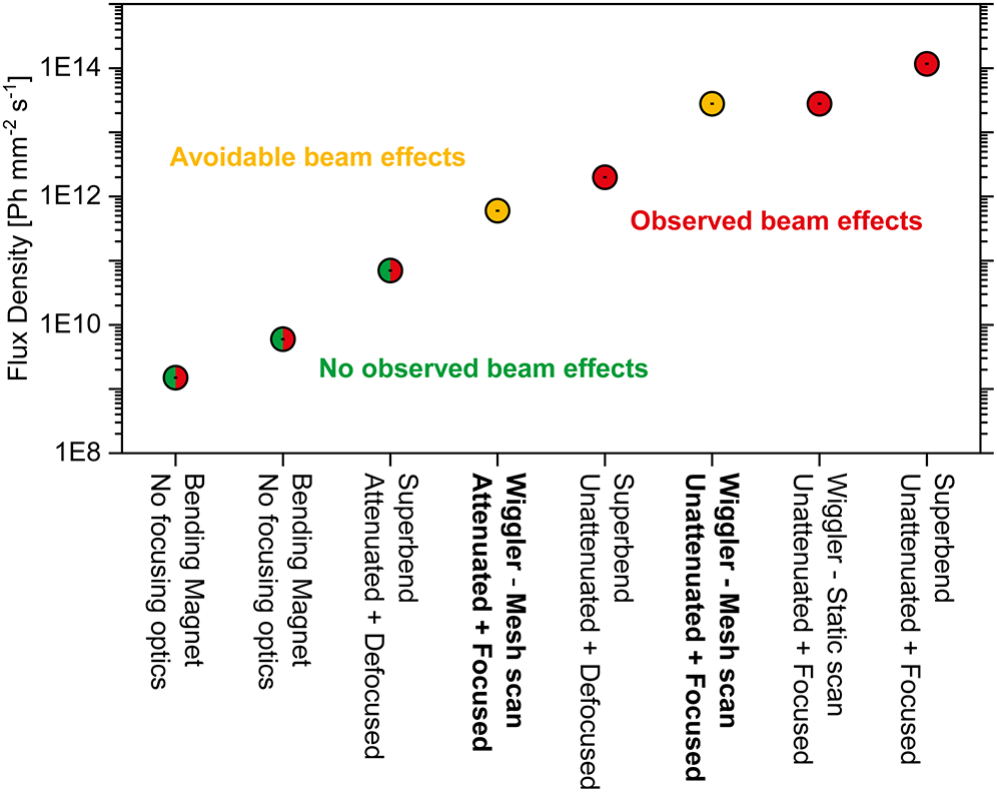
A comparison of the flux densities (at ∼9 keV) applied in present and prior^36^ beam effect studies using the beam-induced reduction of Cu­(II) to Cu­(I) under a CH_4_ atmosphere and the beam-induced oxidation of Cu­(I) to Cu­(II) under an H_2_O/He atmosphere in Cu/zeolites as a probe reaction. The *x*-axis indicates the experimental setup employed for the given measurements. The experimental setups for which a mesh scan was applied are depicted in bold. The *y*-axis indicates the flux density achieved with the given experimental setup. The experiments where beam-induced effects were witnessed are marked in red, while the experiments where no beam-induced effects were witnessed are indicated in green. Experiments where beam-induced effects were observed, though are avoidable when choosing the mesh scan procedure, are indicated in yellow.

Figure 4 summarizes how variations in flux density will influence if any beam-induced effects will arise for the examined reaction conditions. In the case of the reduction of Cu­(II) to Cu­(I) in Cu/zeolites under a CH_4_ atmosphere, at flux densities above ∼10^11^ Ph mm^–2^ s^–1^ a beam-induced reduction becomes unavoidable if a given spot is continuously illuminated, rendering any meaningful interpretation of the data impossible. This regime is indicated by a red shading in Figure 4. A yellow shading instead indicates where the same flux densities may still be employed to obtain meaningful results, provided that the mesh scan approach outlined in this study is applied. In the case of the flux densities examined below ∼10^11^ Ph mm^–2^ s^–1^, previous results suggest that none to minor beam-induced effects will influence the reduction of Cu­(II) to Cu­(I) during CH_4_ exposure.^36^ This is indicated by a green shading in Figure 4. Therefore, the potential ramifications on data integrity may be minor to none if excessive illumination is avoided.

The effects that a beam may have however clearly extend beyond the alteration of reaction kinetics alone. Such effects are observed under all gas atmospheres that Cu/omega has been exposed to. Under an O_2_ atmosphere, unexpectedly, a reduction of Cu­(II) to Cu­(I) is observed (Section 4, Supporting Information). A potential source of the observed Cu­(II) reduction may be the beam ionizing residual carbonaceous deposits present in the zeolite, or a local heating effect due to the beam resulting in their gasification, and those species in turn yielding a reduction of Cu­(II) to Cu­(I). Residual carbonaceous deposits being able to reduce Cu­(II) to Cu­(I) in Cu/zeolites is a well-known phenomenon.^71^ Exposure of the sample to high flux densities of X-rays may therefore potentially shift the onset of this process to lower temperatures. If this is the correct conclusion for the observed Cu­(II) to Cu­(I) reduction under O_2_, then the onset of this process occurring at much lower temperatures than expected may easily lead to a misinterpretation of the processes occurring in the system.

The effects observed under O_2_ and CH_4_ exposure in the case of Cu/zeolites therefore seem to at least partially result in altered reaction kinetics, which may to a certain degree be due to localized heating. Exposing a Cu­(I)/zeolite to both He and H_2_O vapor at high flux densities instead seems to alter the chemistry occurring in the system, resulting in completely new reaction routes. When applying a static scan at flux densities above ∼10^11^ Ph mm^–2^ s^–1^ under both He and H_2_O vapor, a sharp increase in Cu­(II) at the expense of Cu­(I) is observed. Such a high flux density is therefore unapplicable for these conditions and is indicated by the red coloring in Figure 4. When a mesh scan is instead applied, no change at all in the oxidation state of Cu is observed. This is indicated by the yellow shading in Figure 4. To make matters even more complex, an oxidation of Cu­(I) to Cu­(II) is even witnessed at flux densities below ∼10^11^ Ph mm^–2^ s^–1^ at very similar reaction conditions. If Cu­(I)/zeolites can be oxidized via H_2_O vapor exposure is an ongoing debate in literature.^59,60^ Many factors such as the Cu-loading and Cu-species that results from said loading are suggested to affect the ability of Cu­(I) to be oxidized to Cu­(II) in Cu/zeolites.^72^ The Cu­(I)-species known to primarily be present in Cu/omega are however deemed to not be able to be reoxidized via H_2_O vapor exposure.^61,64,72^ The results of this study clearly point toward that being the correct assertion. Therefore, exposing Cu­(I)/zeolites to H_2_O vapor in the presence of a static beam using a flux below ∼10^11^ Ph mm^–2^ s^–1^ may already suffice to adulterate any results, and is therefore indicated with a red coloring in Figure 4.

Finally, the observations derived from exposure of a Cu/zeolite to H_2_O stand in contrast to what has been typically observed when exposing Cu­(II) to H_2_O in the presence of an X-ray beam. Literature has documented that Cu­(II) in aqueous solution will lead to a reduction of Cu­(II) to Cu­(I),^45,46^ which may be attributed to solvated electrons or other reducing species created by the radiolysis of H_2_O or its knock-on reactions.^33^ However, exposing a fully oxidized Cu­(II)/zeolite to H_2_O vapor following an O_2_ exposure instead results in an almost negligible (<1%) increase in the Cu­(I) fraction, as illustrated in Figure S6 (Supporting Information). A potential explanation for the discrepancy between literature and the observed results may therefore stem from the zeolite matrix in which the Cu is hosted. The zeolite lattice itself could be responsible for scavenging any reducing species itself, which would additionally entail that the produced oxidizing species cannot recombine to form H_2_O, and could therefore be available to oxidize Cu­(I) to Cu­(II).

While the degree and type of effects will clearly differ from system to system, the results highlight that special attention must be taken when conducting in situ studies using X-ray illumination in the presence of H_2_O. This is problematic as H_2_O is not just unavoidably present in a range of materials, but also used in a wide range of applications and reactions. H_2_O is for one becoming a more common and feasible solvent used for a range of applications,^73^ which would therefore complicate any investigation of said reactions via X-rays. In situ and operando investigations via X-ray illumination has in recent years emerged as a common characterization technique in, for example, electrocatalysis, where H_2_O is not only a common reactant, but used as the electrolyte itself.^74,75^ More traditionally, X-rays have been used to study thermocatalytic or themocatalysis-adjacent reactions where H_2_O is a common feature. H_2_O is employed as a reactant in a range of catalytic reactions, such as methane-steam reforming^76^ or in the water–gas shift reaction.^77^ Conversely, H_2_O emerges as a product in a range of catalytic reactions, such as the CO_2_ hydrogenation to CH_3_OH or the reverse-water gas shift reactions,^78^ as well as any Fischer–Tropsch^79^ or oxidative dehydrogenation processes.^80^ A further reaction that is an obvious candidate to be highly susceptible to beam-induced alterations in the presence of H_2_O is the NH_3_-SCR reaction via Cu/zeolites, where H_2_O emerges as a product.^55,81,82^ A fair prediction would additionally be to assume that the beam-induced changes to the chemistry of a system will not only pertain to H_2_O, but for example, to NH_3_ as well. Extreme care must therefore be taken in such cases, and such reactions should be extensively probed for any beam-induced alterations prior to making definite statements about the chemistry occurring in such systems.

### 4.2 Strategies for Minimizing Beam Induced Effects

Exposing a sample to less radiation will logically result in less pronounced beam-induced effects. However, many experiments nowadays necessitate a high temporal resolution or long measurement times, resulting in the exposure of a given material to high amounts of radiation. The results in this work emphasize that a material can be exposed to very high flux densities if a spot is not re-exposed to the beam. Using a mesh scan, the flux density without crucial beam-induced effects is approximately 3 orders of magnitude higher than the flux density previously successfully employed for the same probe reaction without significantly attenuating the beam itself.

A mesh scan may therefore become a necessary feature for certain types of materials whenever the measurements do not allow for the beam to be defocused and attenuated to the required degree. A balance between the desired temporal resolution, the sample bed length and spot size must be found that works for each sample and experiment. At high flux densities very short exposure times clearly suffice to collect high quality data (Figure S4, Supporting Information). The inclusion of a dead-time via a fast-shutter can help minimize unnecessary beam exposure while preserving the desired temporal resolution. To further guarantee uncompromised results, the workable distance between spots will need to be examined for every material and reactant applied to avoid any spillover of beam-induced effects into sample areas remaining to be examined. This will differ significantly depending on the material’s characteristics, as well as on the reactants and products. An added benefit of such a methodology is that a study of any changes along the sample bed will be performed simultaneously, adding further insights into a materials’ behavior under reaction conditions.^83–85^

A suggested measurement procedure would be as follows: (i) perform a scan at the highest achievable flux density, (ii) close the shutter and move to the next position (iii) incorporate a dead-time adjusted for bed length, beam size, and total measurement time, and (iv) perform the next scan. This approach, at least for the systems discussed in this work, prevents beam-induced alterations to the data, ensuring chemically reliable and high-quality results.

This outlined methodology should not be seen as a guarantee for completely avoiding beam-induced alterations to the measurement. To guarantee chemically sound data therefore, each system or reaction probed via X-rays should be examined in its own right to see if any undesired effects due to illumination may arise, and how those effects may be avoided. For example, a prerequisite for the application of the mesh scan approach is spatial uniformity of the sample, as well as the lack of a thermal gradient, across the entirety of the probed bed.^68^ In scenarios where these prerequisites cannot be guaranteed, a defocused and attenuated beam may instead be the more reasonable course of action.

For further strategies to avoid beam-induced effects, it may be valuable to additionally consider research fields not limited to materials science and catalysis that employ synchrotron radiation to investigate samples where beam damage must be avoided at all circumstances, such as cultural heritage materials. We therefore direct the reader toward the following research works, which provide an overview not only of potential beam-induced effects, but also a discussion of potential strategies to identify the presence of and how to avoid such adverse effects.^11,13,68^ As beam-induced effects may manifest in a multitude of ways, broadening one’s perspective to include strategies developed for completely different types of materials will be highly beneficial for minimizing such effects during measurement.

## 5 Conclusions

This work underscores the deleterious and misleading effects that may arise by exposing samples to X-ray beams of a high flux density, and demonstrates that these adverse effects may already be present at much lower flux densities than previously expected—in the case of Cu/zeolites. More importantly, this work highlights that completely new reaction pathways may be accessed in the presence of an X-ray beam under certain reactive environments. By implementing a mesh scan across the sample bed, these effects may however be determined and mitigated, thereby delivering results that accurately reflect the material’s intrinsic properties free from any beam-induced artifacts.

Nevertheless, sweeping generalization regarding the absence of beam-induced effects across different materials and catalysts need to be avoided—each system must be individually evaluated, particularly when exposed to potentially reactive conditions. As synchrotrons and beamlines continue to advance, these deleterious effects will become increasingly prevalent and pronounced. The presented approach should therefore serve as a framework to probe for any beam-induced effects, to arrive at measurement conditions that can achieve reliable results while still fully leveraging the superior flux density, smaller beam sizes, faster scan times, and resulting superior data quality offered by next-generation synchrotron facilities.

## Supplementary Material



## Data Availability

Data for this article, including the normalized spectra used for all figures as well as the references used to perform the LCF, are available at Zenodo at [10.5281/zenodo.15000487].

## Notes

Section 1: References employed for linear combination fitting (LCF). Section 2: Further evaluation of beam induced effects during CH_4_ exposure: variation of flux densities and temperatures, evaluation of spectral quality as a function of flux density and exposure time. Section 3: Further evaluation of beam induced effects during H_2_O exposure. Section 4: Evaluation of Beam induced effects during O_2_ exposure (PDF)
