# Supplementary material for: Identifying and Mitigating Adverse X‑ray Induced Effects in Operando Spectroscopic Studies of Copper Exchanged Zeolites
Source: J Phys Chem C Nanomater Interfaces. 2025 Nov 26;129(50):22191–201. doi: 10.1021/acs.jpcc.5c06740 (PMC12720238; doi:10.1021/acs.jpcc.5c06740)
Supplement: Supplementary file 1 [file jp5c06740_si_001.pdf]

Identifying and Mitigating Adverse X-ray Induced Effects in Operando Spectroscopic Studies of Copper Exchanged Zeolites

Supporting Information

Johannes Wieser[1], Mark A. Newton[2], Przemyslaw Rzepka[2], Paul M. Leidinger[3],Mahesh Ramakrishnan[4], Justus Just[4], Jeroen A. van Bokhoven [1,5,*]

1 Department of Chemistry and Applied Biosciences, Institute for Chemical and Bioengineering, ETH Zurich, 8093 Zürich, Switzerland

2 Department of Structure and Dynamics in Catalysis, J. Heyrovsky Institute of Physical Chemistry, Dolejškova 2155/3, 182 23 Prague 8, Czech Republic

3 Interdisciplinary Nanoscience Center, Aarhus University, Gustav Wieds Vej 14, 8000 Aarhus C, Denmark

4 MAX IV Laboratory, Fotongatan 2, 224 84 Lund, Sweden

5 Center for Energy and Environmental Science, Paul Scherrer Institute (PSI), 5232 Villigen, Switzerland

*Corresponding author email: jeroen.vanbokhoven@chem.ethz.ch

1. References employed for the Linear Combination Fitting (LCF)

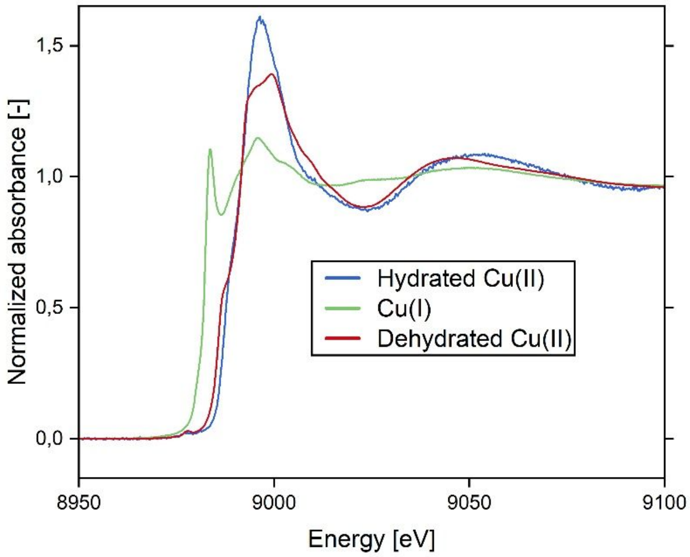

Figure S1: Cu references of Cu-omega employed for the LCF of the different stages of the CH_4-to-CH_3OH (MtM) conversion.

Figure S1 depicts the derived internal standards for hydrated Cu(II), Cu(I) and dehydrated Cu(II) used for the LCF. The reference for hydrated Cu(II) was collected at room temperature. Ten spectra were averaged for the hydrated Cu(II) reference. The sample was then heated to 450 °C under an O_2 atmosphere. The sample was kept at these conditions for 30 minutes, at which point 145 spectra were collected. These were averaged and represent the reference for dehydrated Cu(II). After a He gas purge, the sample was exposed to CH_4, with the temperature kept constant at 450 °C. After one hour, and no changes being witnessed anymore in the spectra, eight spectra were collected. These were averaged and represent the reference for Cu(I).

2. CH_4 Exposure

2.1 Comparing the Rate of Cu(II) to Cu(I) Reduction Across Varying Flux Densities and Temperatures

Figure 2 depicts a comparison of the change of the Cu(I) fraction as a function of time at various flux densities and temperatures. Prior to the exposure to CH_4, all materials were oxidized in O_2 to guarantee that Cu would be present in an oxidation state of +2.

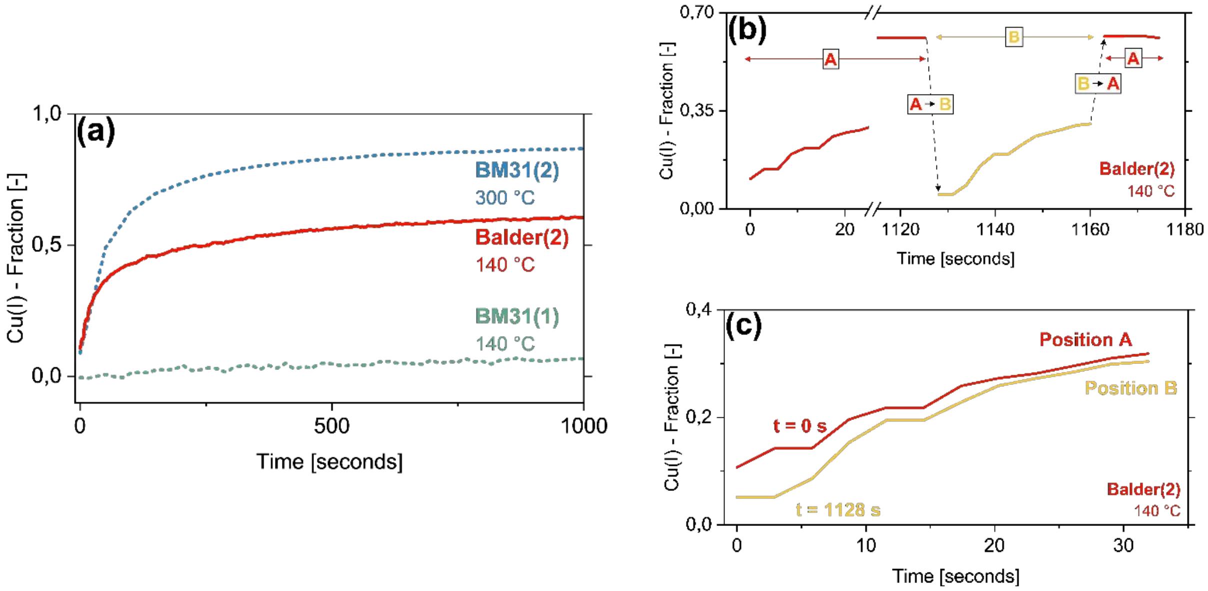

Figure S2: Kinetics of Cu(I) formation in Cu/omega with a focussed unattenuated beam (Balder(2) – Table 1) under a CH_4 atmosphere of 1 bar at 140 °C, using a two second scan time. The Cu/omega sample was previously exposed to an O_2 atmosphere at 300 °C, resulting in primarily dehydrated Cu(II) being present in the system pre CH_4 exposure. **(a)** Comparison of results obtained for Cu/omega at high flux beamline (Balder(2) – red solid line), versus a Cu/omega sample at 300 °C (BM31(2) – blue dotted line)[1], and a Cu/mordenite sample at 140 °C (BM31(1) – cyan dotted line)[2], both obtained at a low flux beamline during CH_4 exposure. The labels correspond to the flux densities applied and listed in Table 1 of the main text. Experiments performed during these studies are indicated in full lines, while results from prior work are indicated in dotted lines.[1,2] In all cases a three-component fit is applied; **(b)** The change in the Cu(I) fraction of Cu/omega-A when moving to a position on the sample bed previously unexposed to the beam with a focussed unattenuated beam (Balder(2) – Table 1). The Cu(I) fraction at position A, after moving to position B, as well as after returning to position A are depicted. The red line indicates the change in the Cu(I) fraction observed at position A, while the yellow line indicates the change in the Cu(I) fraction observed at position B; **(c)** The initial rates of Cu(I) formation at both position A and position B. In the case of Position A, the Cu(I) fractions are recorded for the first 30 seconds of CH_4 exposure. Position B depicts the first 30 seconds after a movement to said position.

Figure S2a depicts the increase in Cu(I) as a function of time at varying X-ray doses and temperatures under a CH_4 atmosphere at 1 bar. At 140 °C and a focussed unattenuated beam (Balder(2) – Table 1), 40 % of all Cu in the system has reduced from Cu(II) to Cu(I) after ~ 300 seconds (Figure S2a – Balder(2)). This rapid increase in Cu(I) over such a short timeframe is in stark contrast to the results obtained at a low flux beamline at 140 °C for Cu/mordenite (Figure S2a – BM31(1)), where the rate of Cu(II) reduction to Cu(I) is significantly less pronounced.[2] The initial rate of Cu(I) formation observed in this case is more comparable to the rate witnessed at 300 °C at a low flux beamline (Figure S2a – BM31(2)), where, under the applied conditions, the contribution of the beam to the Cu(I) formation rate is assumed to be low to negligible.[1] The O_2 exposure conditions (300 °C, 30 minutes) that precede the sample's exposure to CH_4 are identical for both BM31(2) and Balder(2). The source of the divergence in final Cu(I) values at these flux densities must therefore occur under CH_4. This suggests that the mechanism of Cu(II) reduction to Cu(I) by CH_4 may be different when illuminated by a beam of high flux density, instead of the beam solely causing a localized heating in the irradiated volume.

A movement of 200 μm along the bed after ~ 20 minutes of CH_4 exposure to a region previously unexposed to the beam (from position A to position B) reveals that the Cu(I) fraction at the unexposed position (position B) remains

unchanged to pre- CH_4 exposure values (Figure S2b). The first spectrum recorded at position B is therefore a validation that a Cu(II) reduction under a CH_4 atmosphere at 140 °C unilluminated by the X-ray beam in actuality progress very slowly, if at all. The rates of Cu(I) formation under X-ray beam exposure at both positions are very similar (Figure s2c). These results underline that exposing Cu/omega to a beam of high flux density (e.g. Balder(2) – Table 1) under a CH_4 atmosphere will cause a rapid increase in the Cu(I) fraction. This is further verification of the identical adverse effects observed at other synchrotron facilities when a Cu/zeolite is exposed to a high flux density.[2]

A return to position A shows that the Cu(I) fraction remains unchanged to the levels preceding a movement along the sample bed (Figure S2b; 1120 s vs 1170 s). On these timescales, no re-oxidation of Cu(I) to Cu(II) is observed, suggesting that the change caused to the system by the high flux density may be irreversible without exposure to a suitable oxidant. An external influence, e.g. an oxidative atmosphere, is needed to reverse the beam-induced effects by re-oxidizing Cu(I) to Cu(II).

2.2 Cu(II) to Cu(I) Reduction Under Typical Reaction Conditions

As a demonstration of feasibility of the mesh scan technique, a cycle using typical reaction conditions employed in the MtM conversion via Cu/zeolites was performed.[3,4] The flux density was reduced by ~ 98 % (Balder(1) – Table 1), which is, however, still one order of magnitude higher than the values previously suggested to cause no adverse effects in the case of Cu/zeolites.[2] Ten seconds were allocated for a XAS scan, with ten further seconds used to measure the diffraction pattern (at 8,97 keV and 12 keV), equating to a total exposure time of 20 seconds per position. 114 separate points were examined across the sample bed. Different oxygen looping conditions were used. The results are depicted in Figure S3.

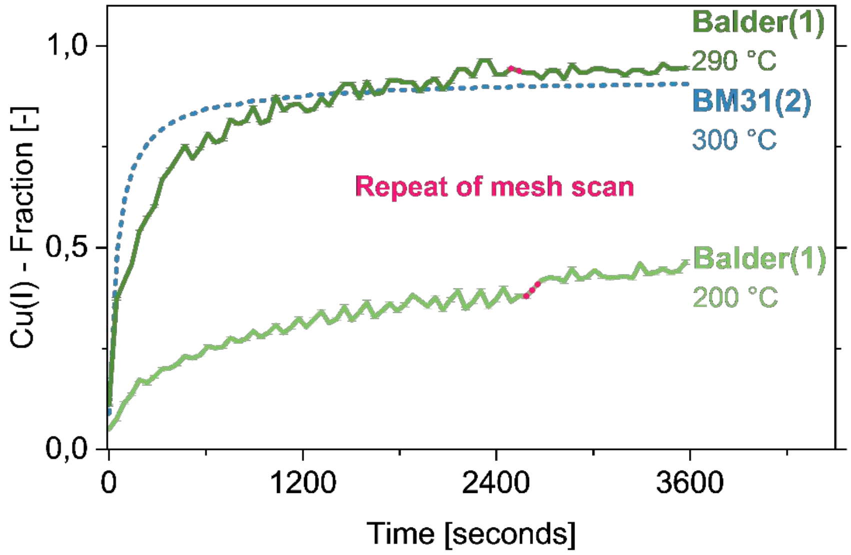

Figure S3: The change in Cu(I) fraction at different temperatures as a function of time at both high flux density (Balder(1) – Table 1) and low flux density (BM31(2) – Table 1) beamlines. The high flux density experiment was conducted using a mesh scan to avoid a beam-induced reduction of Cu(II) to Cu(I) (Balder(1) – 290 °C and Balder(1) – 200 °C). The low flux experiment was conducted using a static scan (BM31(2) – 300 °C). 114 separate spots were examined for both temperatures during the high flux experiment (Balder(1) – 290 °C & Balder(1) – 200 °C).The pink dotted lines indicate when a mesh scan is repeated, e.g. the movement from pos. 114 to pos. 1, as depicted in Figure 3a. For the spectra and conditions collected at the high flux beamline, two scans were averaged, while in the case of the low flux spectra the scans are un-averaged. Experiments performed during these studies are indicated in full lines, while experiments from previous work are indicated in dotted lines.[1] The labels correspond to the flux densities applied and listed in Table 1 of the methods section of the main text.

Figure S3 depicts a change in the Cu(I) fractions over time using different cycling conditions. One cycle was performed using a high-temperature activation procedure under O_2 at 450 °C, followed by an exposure to CH_4 at

200 °C (Balder(1) – 200 °C). A further cycle was conducted with the O_2 and CH_4 exposure steps performed isothermally at 290 °C (Balder(1) - 290 °C). Both these cycling procedures are typically employed not only in the case of Cu/omega[1,3,5,6], but also in the case of other Cu/zeolites examined in the stepwise MtM conversion.[7–9] In both cases a mesh scan was used. Data collected at 300 °C (BM31(2) - 300 °C) using an isothermal looping approach at a low flux beamline and a static scan are depicted as well. Figure S3 shows that the rates of Cu(I) formation increase as a function of temperature, and not as a function of beam dose. The final Cu(I) values achieved differ as well. A fair assumption is that the final Cu(I) value at 290 °C should be similar to the values observed at 300 °C without beam-induced effects. The final value achieved at 290 °C (Balder(1) – 290 °C) is however higher than at 300 °C (BM31(2) – 300 °C). If the source of this variation is due to the sample batch (Cu/omega-A vs Cu/omega-B, see section 2.1 Material Properties in the main text), or if this variation is due to the beam dose, is difficult to discern. However, if the beam dose does have a contribution, it is not very pronounced. After ~ 2500 seconds, when the mesh scan is repeated, no stepwise increase in Cu(I) is observed. At 200 °C, a slight increase in the Cu(I) fraction is observed (Balder(1) – 200 °C). The effect of the beam on Cu(II) reduction to Cu(I) will of course be less significant when most Cu is already present in a reduced form. However, a beam-induced increase in the Cu(I) fraction, even at these drastically reduced flux densities (Balder(1) vs. Balder(3) – Table 1) is still observed. These results, recorded under typical oxygen looping cycling conditions, once again highlight that to ensure chemically sound and interpretable data, a spot should only be examined once when the flux density cannot be sufficiently reduced to levels where no interference is guaranteed.

2.3 Change in Spectral Quality as a Function of X-Ray Dose and Exposure Time

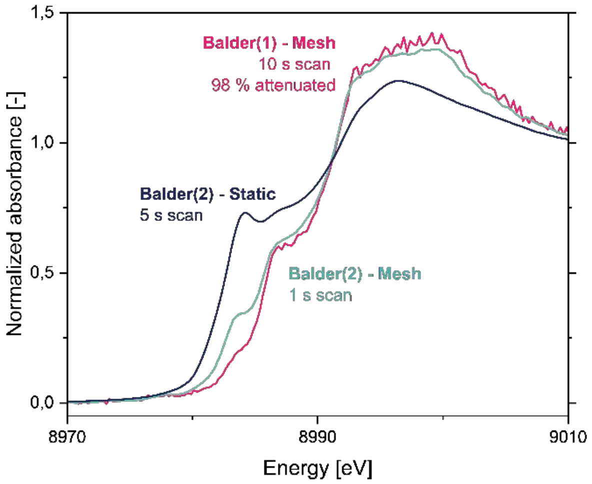

Figure S4: Final X-ray absorption spectra recorded for Cu/omega under a CH_4 atmosphere at 140 °C. Prior to CH_4 exposure, the sample was activated and oxidized under an O_2 atmosphere at elevated temperatures. The labels correspond to the flux densities applied and listed in Table 1 of the Methods section.

Figure S4 presents a comparison of the final spectra recorded during CH_4 exposure at 140 °C using various scan setups. As expected, the scan attenuated by 98 % yields the spectrum with the poorest signal-to-noise ratio (Balder(1) – Mesh). These expected findings highlight that a higher flux density will result in a higher degree of spectral quality. A balance between the applied flux density and avoiding any beam-induced effects is therefore necessary, which becomes more simple when applying the mesh scan.

3. He and H_2O vapor Exposure

3.1 Effect of He and H_2O vapor Exposure on Cu(I)/omega

Figure S5 depicts the change in the Cu(I) fraction as a function of time under initially a He atmosphere, followed by the addition of H_2O vapor (30 mbar) into the reactor feed. The change in all three internal standards (Figure S1) used for the LCF analysis as a function of time under both a He and H_2O vapor atmosphere of the results depicted in Figure 3 (Balder(3) – Static).

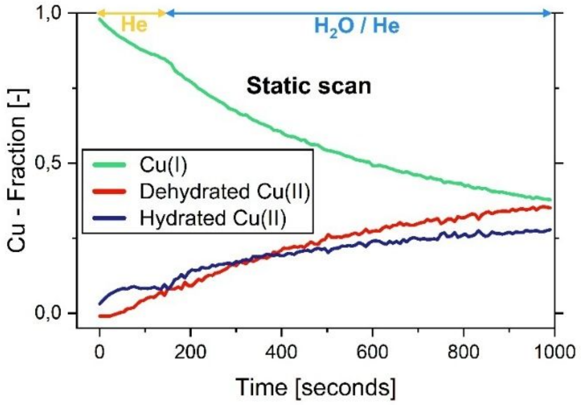

Figure S5: Change in Cu-fractions, initially under a He atmosphere, followed by the additional introduction of H_2O vapor, as a function of time at 290 °C. A three-component fit for the LCF is used. Initially under a He atmosphere, followed by the additional introduction of H_2O vapor, as a function of time at 290 °C. The beam conditions applied are reported in the methods section as Balder(3).

3.2 Effect of H_2O vapor Exposure on Cu(II)/omega

Figure S6 depicts the change, or the lack thereof, in all three internal standards (Figure S1) used for the LCF analysis as a function of time during H_2O vapor atmosphere.

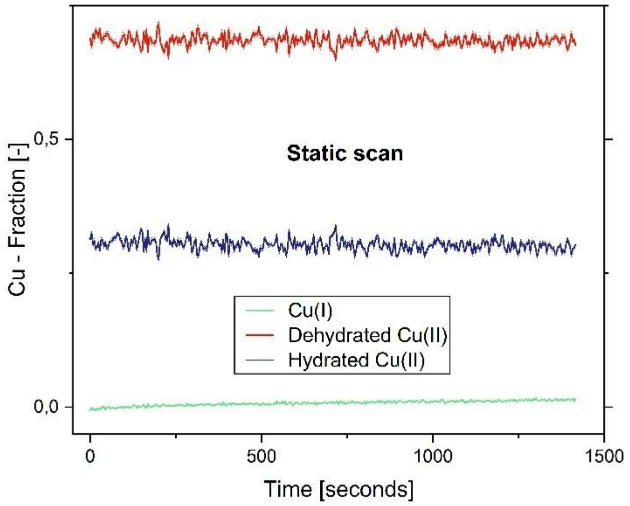

Figure S6: Change in the Cu-fraction, under a flow of He with the addition of 30 mbar H_2O vapor, as a function of time at 290 °C. Prior to the samples exposure to H_2O vapor, the sample was exposed to O_2 at 450 °C. A static scan is used. A three-component fit for the LCF is used. The beam conditions applied are reported in the methods section as Balder(3).

Figure S6 highlights that when exposing Cu(II)/omega to H_2O vapor, the respective Cu-fractions do not seem to change. An increase in the Cu(I) fraction of 1%, starting from 0%, may be observed. This change seems negligible when compared to the changes observed when Cu(I)/omega was exposed to H_2O vapor at the same flux densities (Figure S5). This stands in contrast to what has been observed for Cu(II) in aqueous solutions, where a reduction of Cu(II) to Cu(I), attributed to species produced by the radiolysis of water (or secondary species produced by these) were suggested to be responsible for the observed reduction.[10,11]

4. O_2 Exposure

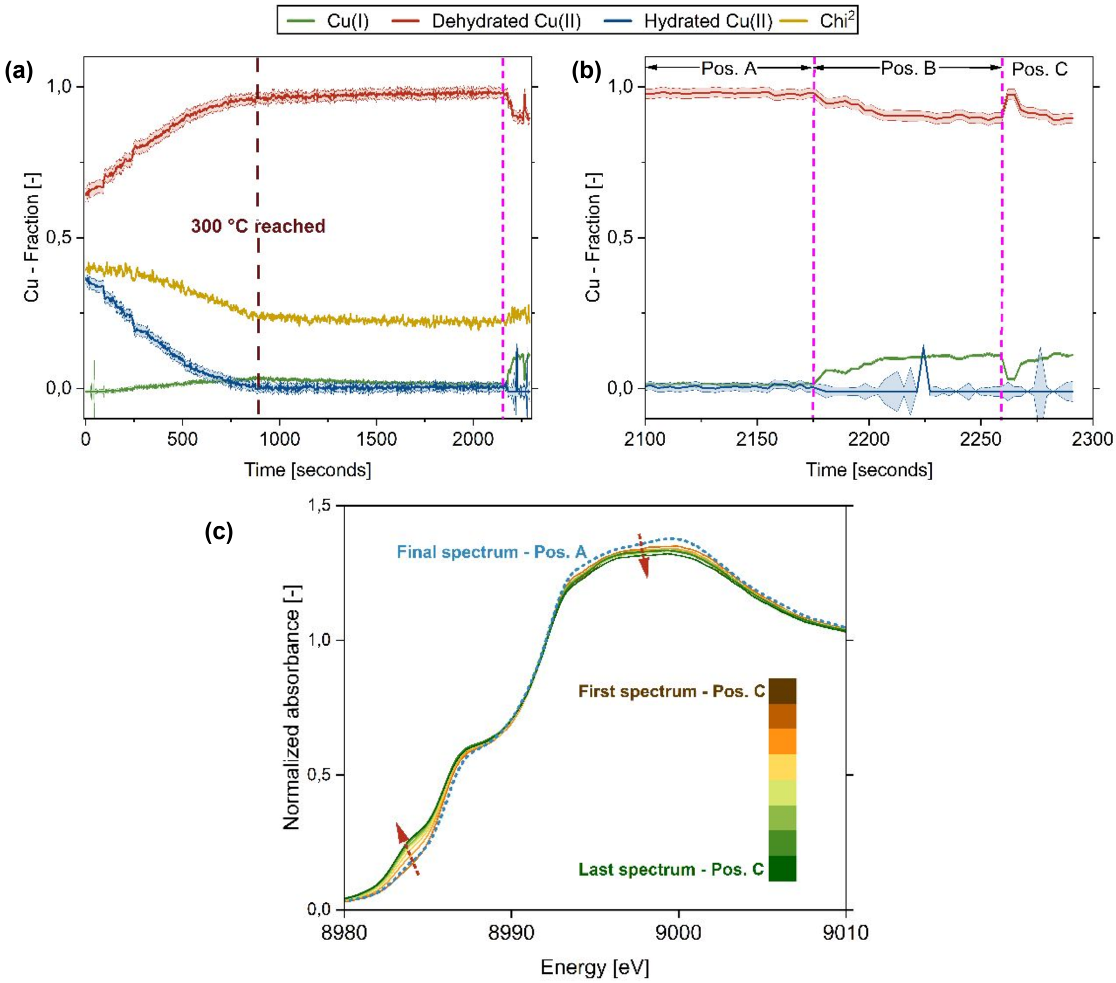

Figure S7: Change in Cu-fractions over time under an O_2 atmosphere during a ramp up to and dwell at 300 °C, using a two second scan time and an unattenuated focused beam (Balder(2) - Table 1 – Methods section) **(a)** Change in Cu-fraction and Chi^2 values. The brown dotted line indicates when 300 °C is reached. The magenta dotted line indicates when a movement across the bed was initialized, as depicted in (b); **(b)** Change in Cu-fraction at three different examined positions. **(c)** Spectra collected during O_2 exposure at position C (Figure S7b). Also included is the final spectrum recorded at position A (Figure S7a-b).

Figures S7a-b depict the effect of exposing the sample to O_2 using an unfocussed and unattenuated beam (Balder(2) – Table 1, Methods section). During the ramp to 300 °C, an increase in both the dehydrated Cu(II) and Cu(I) fractions at expense of hydrated Cu(II) are observed (Figure S8a). While the increase in dehydrated Cu(II) and decrease in hydrated Cu(II) is the typically observed behavior when increasing the temperature under an O_2 atmosphere for Cu/zeolites,[1] an increase in Cu(I) is not as typical. Figure S7a however also shows that following a maximum for the Cu(I) fraction at ~ 800 seconds, the value starts decreasing again. This indicates that whatever process has occurred is reversible. This is in contrast to observations under all other examined atmospheres, where only a change in gas atmosphere is able to regenerate the system to its state pre beam-exposure. That this is not the case under an O_2 atmosphere would suggest that the increase in Cu(I) fraction does not directly stem from an O_2 exposure itself. One potential source may be carbonaceous residues in the zeolite itself from, for example, a synthesis procedure employing organic structure directing agents (OSDA's). An OSDA, TMACl, has in fact been employed in the synthesis of this Cu-omega sample.[5,12] That such carbonaceous residues are able to reduce Cu(II) to Cu(I) when exposed to elevated temperatures is a known phenomenon [13], and may therefore be a reasonable explanation for the observed change in Cu-fractions.

After ~ 2200 seconds, the beam was moved to a new position (Pos. B), as depicted in Figure S8b. A sudden increase in the Cu(I) fraction at the expense of dehydrated Cu(II) is observed. Moving position again (Pos. B to Pos. C) shows the same trend; a sudden increase in the Cu(I) fraction. The spectra recorded at Position C are depicted in Figure S7c. Figure S7c shows that a decrease in the white-line intensity (~ 9000 eV) and an increase in the edge-features around 8985 eV can be observed, which may be attributed to a reduction of Cu(II) to Cu(I). As this observed reduction only starts once exposed to the beam, and after 1000 seconds at 300 °C with no meaningful change in the Cu-fractions being observed (Figure S7a), the beam will obviously be responsible for it. A potential source may therefore be carbonaceous residues present in the zeolite, which without any illumination by a beam would lead to a Cu(II) reduction to Cu(I) at higher temperatures. Cu/zeolites are often pre-treated at temperatures of ~ 450 °C to desorb these carbonaceous deposits. A possible explanation therefore of the observed reduction of Cu(II) to Cu(I) under an O_2 atmosphere at 300 °C may therefore stem from either local temperature effects due to illumination, or possibly by radiolysis of the carbonaceous deposits themselves, leading to species able to reduce Cu(II) to Cu(I).

References

[1] J. Wieser, A. J. Knorpp, D. C. Stoian, P. Rzepka, M. A. Newton, J. A. van Bokhoven, “Assessing the Productivity of the Direct Conversion of Methane-to-Methanol over Copper-Exchanged Zeolite Omega (MAZ) via Oxygen Looping” *Angewandte Chemie International Edition* **2023**, DOI 10.1002/anie.202305140.

[2] M. A. Newton, A. J. Knorpp, J. Meyet, D. Stoian, M. Nachtegaal, A. H. Clark, O. V. Safonova, H. Emerich, W. Van Beek, V. L. Sushkevich, J. A. Van Bokhoven, “Unwanted effects of X-rays in surface grafted copper(II) organometallics and copper exchanged zeolites, how they manifest, and what can be done about them” *Physical Chemistry Chemical Physics* **2020**, *22*, 6826–6837.

[3] J. Wieser, D. Wardecki, J. W. A. Fischer, M. A. Newton, C. Dejoie, A. J. Knorpp, G. Jeschke, P. Rzepka, J. A. van Bokhoven, “Quantifying the Hydration-Dependent Dynamics of Copper Migration and Activity in Zeolite Omega for the Partial Oxidation of Methane” *Angewandte Chemie International Edition* **2024**, e202407395.

[4] M. A. Newton, A. J. Knorpp, V. L. Sushkevich, D. Palagin, J. A. van Bokhoven, “Active sites and mechanisms in the direct conversion of methane to methanol using Cu in zeolitic hosts: a critical examination” *Chem. Soc. Rev* **2020**, *49*, 1449–1486.

[5] A. J. Knorpp, Direct Conversion of Methane to Methanol over Copper-Exchanged Zeolite Omega (MAZ), ETH Zürich, **2019**.

[6] A. J. Knorpp, A. B. Pinar, M. A. Newton, V. L. Sushkevich, J. A. van Bokhoven, “Copper-Exchanged Omega (MAZ) Zeolite: Copper-concentration Dependent Active Sites and its Unprecedented Methane to Methanol Conversion” *ChemCatChem* **2018**, *10*, 5593–5596.

[7] A. J. Knorpp, M. A. Newton, S. C. M. Mizuno, J. Zhu, H. Mebrate, A. B. Pinar, J. A. Van Bokhoven, “Comparative performance of Cu-zeolites in the isothermal conversion of methane to methanol” *Chemical Communications* **2019**, *55*, 11794–11797.

[8] P. Tomkins, A. Mansouri, S. E. Bozbag, F. Krumeich, M. B. Park, E. M. C. Alayon, M. Ranocchiari, J. A. van Bokhoven, “Isothermal Cyclic Conversion of Methane into Methanol over Copper-Exchanged Zeolite at Low Temperature” *Angewandte Chemie International Edition* **2016**, *55*, 5467–5471.

[9] J. W. A. Fischer, A. Brenig, D. Klose, J. A. van Bokhoven, V. L. Sushkevich, G. Jeschke, “Methane Oxidation over Cu2+/[CuOH]+ Pairs and Site-Specific Kinetics in Copper Mordenite Revealed by Operando Electron Paramagnetic Resonance and UV-Visible Spectroscopy” *Angewandte Chemie International Edition* **2023**, DOI 10.1002/anie.202303574.

[10] J. G. Mesu, A. M. Beale, F. M. F. De Groot, B. M. Weckhuysen, “Probing the influence of X-rays on aqueous copper solutions using time-resolved in situ combined video/X-ray absorption near-edge/ultraviolet-visible spectroscopy” *Journal of Physical Chemistry B* **2006**, *110*, 17671–17677.

[11] J. G. Mesu, A. M. J. Van Der Eerden, F. M. F. De Groot, B. M. Weckhuysen, “Synchrotron radiation effects on catalytic systems as probed with a combined in-situ UV-Vis/XAFS spectroscopic setup” *Journal of Physical Chemistry B* **2005**, *109*, 4042–4047.

[12] A. J. Knorpp, A. B. Pinar, C. Baerlocher, L. B. Mccusker, N. Casati, M. A. Newton, S. Checchia, J. Meyet, D. Palagin, J. A. van Bokhoven, "Paired Copper Monomers in Zeolite Omega: The Active Site for Methane-to-Methanol Conversion" *Angewandte Chemie International Edition* **2020**, *60*, 5854–5858.

[13] V. L. Sushkevich, J. A. Van Bokhoven, "Revisiting copper reduction in zeolites: The impact of autoreduction and sample synthesis procedure" *Chemical Communications* **2018**, *54*, 7447–7450.

[14] A. B. Pinar, P. Rzepka, A. J. Knorpp, L. B. McCusker, C. Baerlocher, T. Huthwelker, J. A. Van Bokhoven, "Pinpointing and Quantifying the Aluminum Distribution in Zeolite Catalysts Using Anomalous Scattering at the Al Absorption Edge" *J Am Chem Soc* **2021**, *143*, 17926–17930.
